# Ratooning as a management strategy for lodged or drought‐damaged rice crops

**DOI:** 10.1002/csc2.20007

**Published:** 2020-01-29

**Authors:** Rolando O. Torres, Mignon A. Natividad, Marinell R. Quintana, Amelia Henry

**Affiliations:** ^1^ Systems Physiology Cluster, Strategic Innovation Platform International Rice Research Institute Los Baños Laguna 4031 Philippines

## Abstract

Rice (*Oryza sativa* L.) plants have the ability to develop ratoon tillers if the terminal growing point is lost, such as when the panicle has been aborted, matured, or harvested. We examined postharvest and midseason ratooning as management strategies for damaged rice crops, both in irrigated and rainfed conditions. Genotypic variation was observed in terms of postharvest ratoon tillering, midseason ratoon crop growth after lodging, and midseason ratoon crop growth after drought stress. The genotypic variation in postharvest ratoon tillering was related to stem carbohydrate levels at the time of main crop harvest and was affected by soil moisture levels at the time of main crop harvest. Drought‐tolerant varieties did not consistently show improved ratoon crop growth. After lodging, cutting stems at a height of 30 cm produced the highest numbers of ratoon tillers, and the contribution of the ratoon crop to the total harvestable grain yield was highest when the ratoon crop was initiated at earlier growth stages. The highest ratoon grain yields recovered from lodged crops ranged up to 3.58 t ha^−1^. Total grain yield after drought was improved by trimming the leaves and panicles only in certain conditions and did not appear to be correlated with stem carbohydrate levels. These results suggest that management strategies may be recommended to farmers that exploit the ratooning ability of rice for improved recovery after midseason crop damage.

**Abbreviations**
DAPE: days after panicle emergence
DAS: days after sowing
DS: dry season
TTZ: tetrazolium
WS: wet season

## 1 INTRODUCTION

Rice (*Oryza sativa* L.) plants have the ability to develop tillers and regrow from the stem nodes into a ratoon if the terminal growing point is cut or lost, such as when the panicle has been aborted, matured, or been harvested. Cultivation of the resulting ratoons as a crop is an old technology known to farmers in many rice‐growing regions (Krishnamurthy, 1988; Parago, 1963), but it has not gained widespread application because it does not always guarantee an economic yield. Given that the full labor and material costs of cultivating a ratoon crop could be 70% less than that of a full‐duration rice crop (Flinn & Mercado, 1988), optimizing ratooning as a rice crop management strategy could provide significant economic benefits to resource‐poor farmers. Most research on ratooning has been conducted on irrigated rice, but ratooning is also a potential alternative to planting a second rice crop in rainfed systems, since it requires less water input and matures in shorter duration than the main rice crop.

Ratooning is conventionally practiced after harvest of the main crop when ratoon tillers arise. Recently, ratooning is being emphasized more heavily in research on physiology and management strategies for reduced‐input practices in rice production (Peng, 2017), and ratooning has been the key component in the development of perennial rice (Samson et al., 2018; Zhang et al., 2017). In a review of 126 published reports from 1942 to 1982, Chauhan, Vergara, and Lopez (1985) estimated rice ratoon crop yield to range from 0.1 to 8.7 t ha^−1^. Das and Ahmed (1982) and Khrishnamurthy (1988) claimed that ratoon crop yield can exceed 2 t ha^−1^ or >50% of its main crop yield, and it could even be 140% of the main crop yield (Gopala Reddy & Mahadevappa, 1988). However, postharvest ratooning in general has not yet been commonly adopted in either small‐ or commercial‐scale rice farming due to its variable and usually low grain yields (Andrade, Amorim Neto, de Oliveira, & Fernandes, 1988). Long time to maturity can be a constraint to ratooning by increasing the likelihood of ratoon crops being exposed to unfavorable climatic conditions. Chauhan et al. (1985) remarked that the lack of acceptance of rice ratooning by commercial farmers is due to a lack of good ratooning varieties, uneven maturity, disease and insect problems, lack of location‐specific cultural practices, inferior grain quality, and lack of assured return from investment. Therefore, more research is needed to better harness the ratooning ability of rice as a technology that can be useful to farmers.

One major constraint to rice production that ratooning might help mitigate is lodging, which may occur in a small portion of a plot but can expand throughout the whole field or even the region especially when caused by a typhoon (Boschetti et al., 2015). Rice crops are more susceptible to lodging at later growth stages when the internodes have elongated and the panicles have developed. However, any rice variety would be susceptible to lodging with heavy rains, strong winds, or a combination of both. The result could be heavy yield loss (Blanc & Stobl, 2016), or the crop could be abandoned as a total failure. Since the prospects from ratooning are expected to be higher when carbohydrates still abound in the stubbles and roots (Krishnamurthy, 1988), rice crops that are damaged by lodging—especially at early reproductive stage when stem carbohydrate levels are still high—could potentially be salvaged by ratooning.

Another major constraint in rice production is drought, especially in rainfed and poorly irrigated areas (O'Toole & Chang, 1979). At least 23 million ha of rainfed rice area in Asia are estimated to be drought prone, and drought is becoming an increasing problem even in traditionally irrigated areas (Pandey, Bhandari, & Hardy, 2007). Reduction in grain yield is particularly more serious if drought occurs during reproductive development (O'Toole, 1982; Pantuwan, Fukai, Cooper, Rajatasereekul, & O'Toole, 2002; Saini & Westgate, 2000). Recent development of drought‐resistant rice varieties (e.g., Anantha et al., 2016) may provide more opportunities for ratoon crop cultivation by taking advantage of the limited residual water after the main rice crop. However, the relationship between drought resistance and ratooning ability is not known. Furthermore, pruning of panicles stimulates the development of dormant tiller buds (Hanada, 1993) and panicle abortion due to drought stress may have a similar effect to panicle pruning; we have observed that ratoon tillers can arise when soil moisture levels increase after severe drought. This response implies that with proper management, the ratooning trait may be exploited to increase harvest during drought years.

This study was conducted to characterize rice ratoon growth in the context of mitigating two common abiotic problems affecting rice crops: lodging and drought stress. Our aims were (i) to characterize genetic variation in rice ratooning ability, (ii) to understand the relationship between drought resistance and ratooning ability of rice, and (iii) to test crop management strategies for optimization of ratoon growth in the case of lodged or drought‐damaged rice crops. We hypothesized that genotypic variation in ratooning ability might be related to stem carbohydrate levels and/or root viability at the time of harvest. To test these hypotheses, as well as potential crop management strategies, we evaluated both postharvest and midseason ratooning in two sets of genotypes varying in either drought response or ratooning ability, applying a range of cutting treatments of the matured or damaged tissue.

## 2 MATERIALS AND METHODS

**Figure 1:**
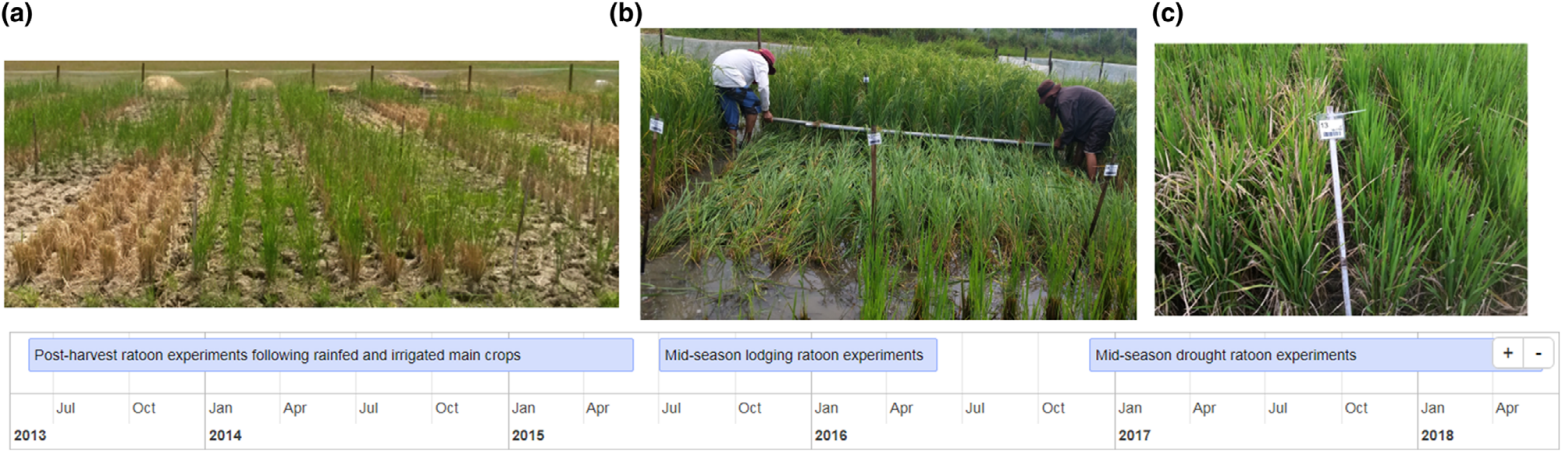
Timeline of experiments conducted in this study. (a) Postharvest ratoon experiments were conducted on drought‐stressed or irrigated main crops to evaluate the genotype and cutting‐height effects on ratoon tillering. (b) Midseason lodging treatments at a range of growth stages were conducted to evaluate ratoon crop growth at increasing cutting heights. Galvanized iron pipes were dragged over the standing crops to impose lodging treatments. (c) Midseason drought stress treatments were imposed to evaluate genotype and main crop trimming treatment effects on ratoon crop productivity. Trimming treatments of the main crop were applied based on the drought response of each genotype, that resulted in plots with and without panicle abortion due to drought

Three sets of experiments were conducted in this study to evaluate (i) postharvest ratooning after growth of a main crop under either drought or well‐watered conditions, (ii) ratoon growth and yield after midseason lodging in well‐watered conditions, and (iii) ratoon growth and yield after severe reproductive‐stage drought stress (Figure 1), with rice genotypes included that were common to each of the three experiments.

The agronomic practices for main crop establishment were common to all experiments in this study. The experiments were conducted in lowland fields with Aquandic Epiaquoll soil at the International Rice Research Institute (IRRI), Los Baños, Laguna, the Philippines (14°10′ N, 121°15ʹ E). The main crops were established by transplanting 17‐ to 20‐d‐old seedlings in paddy soil at 25‐cm row and 20‐cm hill spacing with row length of 3 m. Complete fertilizer was applied ∼2 wk after transplanting at a rate of 50 kg ha^−1^ each of N, P_2_O_5_, and K_2_O. Ammonium sulfate was topdressed during panicle initiation stage at a rate of 50 kg N ha^−1^. Main crops under well‐watered treatments were maintained by flood irrigation to a standing water depth of ∼2 cm until about the physiological maturity stage. For the main crops under drought treatments, irrigation was withdrawn starting at least 4 d after N topdressing at about the panicle initiation stage of the earliest‐duration genotype so that drought stress would coincide with the flowering stage. The well‐watered and drought‐stressed treatments on main crops were imposed in separate but adjacent fields at least 10 m apart. Soil moisture conditions were monitored at a soil depth of 30 cm using tensiometers and frequency domain reflectometry (Diviner; Sentek Sensor Technologies). Drought‐stressed main crops were rewatered by flash irrigation on two consecutive days when the soil water potential at 30‐cm soil depth reached about −60 kPa. Grain yields of the main crops were determined from a 1.5‐m^2^ sampling area. Ratooning experiments were established from these various main crop treatments.

Root viability and stem carbohydrate levels were assessed to relate hypothesized physiological mechanisms to ratooning ability across the three sets of experiments. Root viability was determined by tetrazolium (2,3,5‐triphenyltetrazolium chloride, TTZ) staining modified from the protocol described by Sturite, Herkensen, and Breland (2005) to optimize the staining time, incubation temperature, and TTZ concentration that resulted in the strongest color and best contrast between viable and nonviable root samples. Separate root subsamples were used to represent viable and nonviable samples. The nonviable samples were boiled in water for 45 min to inactivate the roots.

After sampling root crowns from the field (described below), roots were cut into about 150 2‐cm‐length segments and placed in a test tube wrapped in aluminum foil to exclude light. In each test tube, 2:1:1 of 0.6% (w/v) TTZ, 0.06 M phosphate buffer, and 0.05% (v/v) Tween 20 were added with an amount just enough to submerge all the root samples in the solution (13–50 ml depending on the volume of the root sample). Root samples were covered and incubated at 32°C for 24 hr. Approximately 50 segments of TTZ‐stained root samples from each test tube were dispersed on a transparent tray in distilled water, covered with a blue plastic sheet, and scanned (Epson Expression 11000XL 3.49). Images were analyzed using color analysis in WinRhizo Pro 2017 (Regent Instruments), in which the percentage of stained roots was determined by the proportion of projected root area that was stained red based on color classes that were set to classify each pixel as “viable” or “nonviable.” In the 2017WS treatment, boiled subsamples were used as a nonviable color control to more accurately select the viable root area.

Stem carbohydrate levels were measured using anthrone reagent according to Conocono, Egdane, and Setter (1998). Three random tillers from one plant in each plot were collected at midmorning as described below, oven dried, and ground. Sugars soluble in ethanol were extracted from a subsample of 200 mg ground tissue, with two technical replicates measured from each sample. Anthrone reagent was added to an aliquot of the extracted soluble sugar solution, boiled at 95°C for 10 min, and immediately cooled in an ice bath for 5 min. The sample was vortex mixed and equilibrated at room temperature prior to absorbance reading at 620 nm (UV‐1800 spectrophotometer, Shimadzu Corporation). The concentration of each sample was determined in reference to a glucose standard curve (dextrose, anhydrous, Fischer Scientific).

### 2.1 Postharvest ratooning after main crop well‐watered and drought treatments

To characterize postharvest ratooning, two sets of genotypes (Supplemental Table S1) were selected from drought screening trials in the 2012 dry season (DS) (reported by Torres & Henry, 2018) based on their contrasting yield under drought (the “drought resistance” set) or their contrasting ratooning ability (the “ratooner” set), which was assessed after harvest of the trial. Separate experiments on postharvest ratooning were conducted on these two sets of genotypes in two consecutive dry seasons and wet seasons (WS) from 2013WS to 2015DS. The main crops were grown under both well‐watered and drought‐stressed treatments except in 2013WS. Ratoon crops were initiated by cutting the main crop to stubble height treatments of 5 and 20 cm at the time of maturity of each genotype. The main crop experiments were laid out in a randomized complete block design with four replicates, and after harvest, each main crop plot was divided into two cutting height treatments in a split‐plot design. Genotypes were arranged as main plots and stubble cutting heights as subplots (four 3‐m rows per subplot). The ratoon crops were grown on residual soil moisture and rainfall after harvest of the main crop, without additional fertilizer application.

Zero or very low levels of grain yields were obtained from the postharvest ratoon crops due to either tungro virus (*Rice tungro bacilliform virus* + *Rice tungro spherical virus*) infestation or very severe drought stress. Therefore, the only postharvest ratoon crop growth measurement obtained was on tillering. Ratoon tiller numbers were counted from six hills per plot at 3 wk and at about 5–7 wk after the stubble cutting.

In the 2014DS experiments, root crowns of three hills per plot of the earliest‐maturing genotypes were excavated using a shovel inserted at about a 10‐cm radius around the base of the plant to a depth of ∼25 cm. Roots were washed from the soil a day after sampling and subjected to TTZ staining to assess root viability as described above. The stubble remaining after harvest (stem tissue) associated with each root crown was ground and analyzed for carbohydrate concentration as described above.

### 2.2 Midseason ratooning: Lodged rice crops

Experiments to characterize ratoon growth of lodged rice crops were conducted in 2015WS and 2016DS. The main crops were established using split plots in a randomized complete block design with three replications, all under well‐watered conditions.

Genotypes IR77959‐35‐1‐5‐3‐2‐1 (drought resistant) and IR80402‐88‐1‐1‐3 (drought susceptible) were planted in combination with four crop stages at which lodging and ratoon growth was imposed (0, 10, 20, and 30 [maturity] d after panicle emergence [DAPE]) as main plots. In the main crops of the lodging experiments, both genotypes flowered at around 85 and 70 d after sowing (DAS) in 2015WS and 2016DS, respectively. Correspondingly, crop lodging treatments comprised the subplots and were applied at panicle emergence stage on both genotypes at 88 and 72 DAS in 2015WS and 2016DS, respectively.

Lodging treatments were imposed by horizontally dragging a pair of 5‐cm galvanized iron pipes over the planted rows (Figure 1) at 0, 10, and 20 DAPE. A 30‐DAPE treatment was included to asses ratoon growth but was allowed to mature without lodging since the crop reached maturity by ∼30 DAPE. Ratoon crops were established by cutting and removing the upper portion of the plant shoots at different stubble heights 3 d after they were lodged. Three stubble cutting height treatments (5, 15, and 30 cm in 2015WS, and 30, 40, and 40 cm with lock‐lodging in 2016DS) comprised the subplots (Supplemental Figure S1). Very few ratoon tillers formed in the stubbles that were cut at the 5‐cm stubble height in 2015WS. For this reason, taller stubble cutting height treatments were used in 2016DS. The lock‐lodging treatment with 40‐cm stubble cutting height evaluated in 2016DS was adapted from the method described by Calendacion, Garrity, and Ingram (1992), in which the stubbles were bent at the base and braided to keep them flat on the soil surface. The lock‐lodging treatment was intended to force the ratoon tillers to grow close to the soil surface and possibly develop new roots.

Grain yield of the main crop was measured from plots that had filled grains at the time of imposing lodging, as well as from plots that were not lodged (the 30‐DAPE treatment). Ratoon crops were allowed to develop in all plots, including those that did not receive a lodging treatment. Ratoon crops were flash irrigated to maintain the soil moisture level around field capacity until harvest.

### 2.3 Midseason ratooning: Drought‐damaged rice crops

Three experiments characterizing ratoon crops after midseason drought were conducted in open fields in 2017DS and 2018DS and using an automated rainout shelter in 2017WS. In 2017WS and 2018DS, staggered sowing based on the flowering duration of each genotype was done in an attempt to synchronize the dates that each genotype entered the reproductive stage. The treatments were laid out using split plots in a randomized complete block design with three replications. The main plots (eight 3‐m rows) composed of selected drought‐tolerant genotypes and some modern high‐yielding cultivars (Supplemental Table S1); these main plots were split into halves with four rows as subplots for trimming treatments. The main crops were subjected to drought by draining the field when the first genotypes entered panicle initiation stage at 59, 51, and 53 d after final sowing in 2017DS, 2017WS, and 2018DS, respectively. The aim of the drought stress treatment was to result in drying of the emerged panicles and for grain filling in those panicles to be aborted. The drought stress was allowed to progress for 51, 36, and 45 d before applying trimming treatments, at which time the soil water potential at a depth of 30 cm had progressed to −57, −52, and −57 kPa in 2017DS, 2017WS, and 2018DS, respectively (Supplemental Figure S2).

Due to differences in phenology and drought tolerance, the level of drought stress experienced among genotypes was variable and the ratooning treatments were adjusted accordingly in each season. Two pairs of trimming treatments were imposed: (i) one subplot was trimmed to a 30‐cm stubble height and the other subplot was uncut; (ii) one subplot was trimmed to a 30‐cm stubble height and the other subplot was cut at the panicle base level in order to harvest the grains.

In 2017DS, only the longest‐maturing (>90 DAS) genotypes DK124 and PSBRc68 showed complete panicle abortion due to drought; the subplots were trimmed to 30 cm or uncut for these two genotypes and trimmed to 30 cm or trimmed to panicle level for the remaining genotypes. In 2017WS, all genotypes showed partial panicle abortion and were trimmed to 30 cm or uncut. In 2018DS, six exhibited aborted panicles in most tillers and were trimmed to 30 cm or uncut, whereas four of the genotypes flowered earlier and were able to escape from severe drought stress and these genotypes were trimmed to 30 cm or panicle level. Immediately after the cutting treatments were imposed, the experiments were flash irrigated to reach a soil moisture level around field capacity, at which the fields were maintained until crop maturity. Grains (if present) were harvested at the time of cutting the main crop and again at the time of ratoon crop maturity.

In 2017WS, root crowns of one hill per plot were sampled for physiological characterization. Root viability was assessed on samples collected 1 d after applying the trimming treatments and rewatering. Stem carbohydrate levels were assessed as described above on samples collected at the end of the drought stress treatment, as well as at 13 and 48 d after applying the trimming treatments and rewatering.

### 2.4 Statistics

Genotypic differences were analyzed per experiment by ANOVA for randomized complete blocked with one‐ to two‐factorial design. Combined ANOVA was performed to analyze the genotypic differences across seasons using the Shapiro–Wilk test for normality, Bartlett's test for homogeneity of variances, and Tukey's honest significant difference test and LSD test for pairwise mean comparison. Pearson product–moment correlation was used to identify the relationship between physiological traits measured. These statistical analyses were performed using R version 3.1.3 (R Core Team, 2016) and Statistical Tool for Agricultural Research (STAR version 2.0.1, IRRI).

## 3 RESULTS

### 3.1 Postharvest ratooning after main crop well‐watered and drought treatments

**Figure 2:**
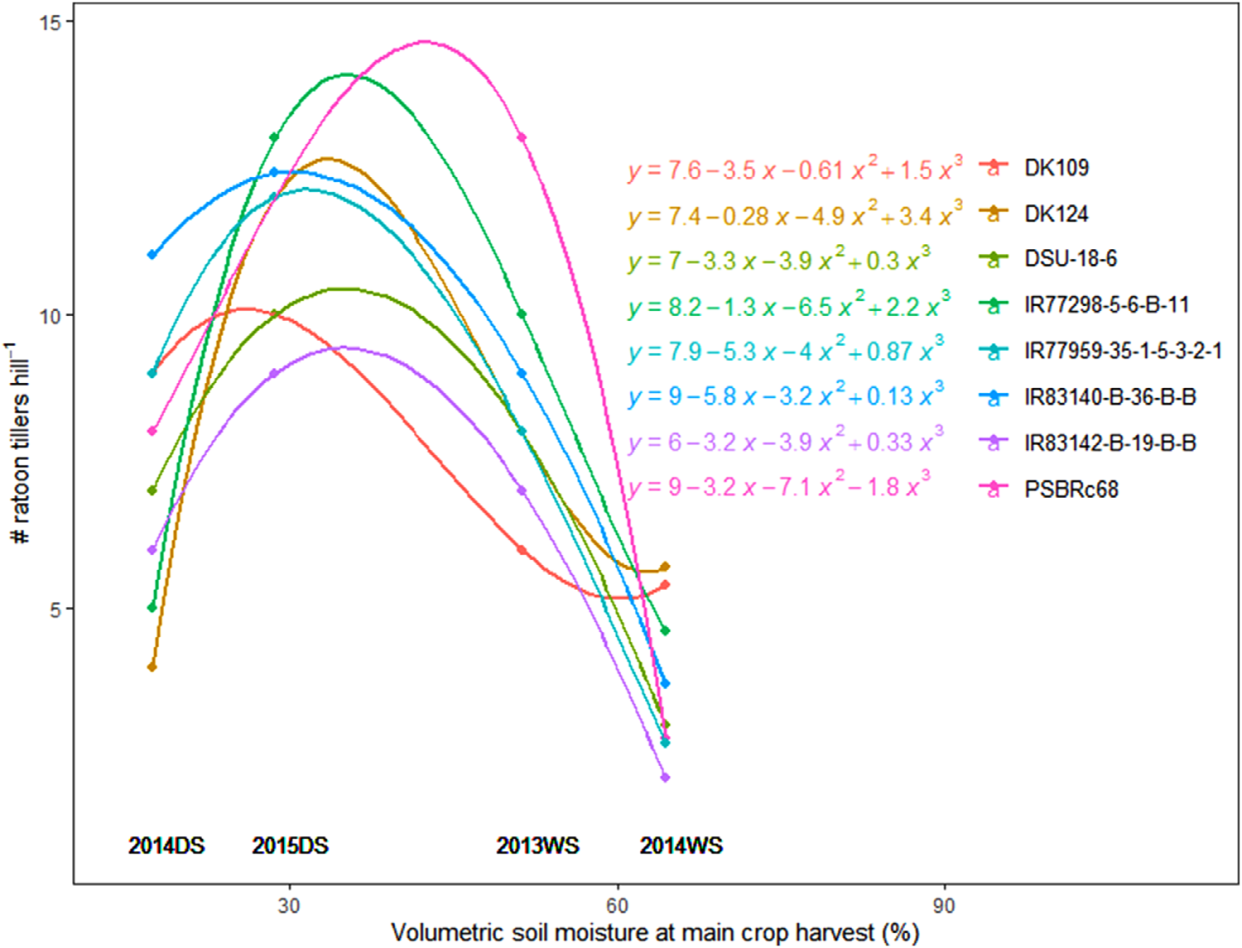
Postharvest ratooning: the maximum number of ratoon tillers per hill as a function of soil moisture (30‐cm depth) at the time of harvest of the main crop (initiation of the ratoon crop) from 2013WS–2015DS. For clarity, genotypes with the highest ratoon tillering are shown from the 20‐cm cutting height treatment. DS, dry season; WS, wet season

**Figure 3:**
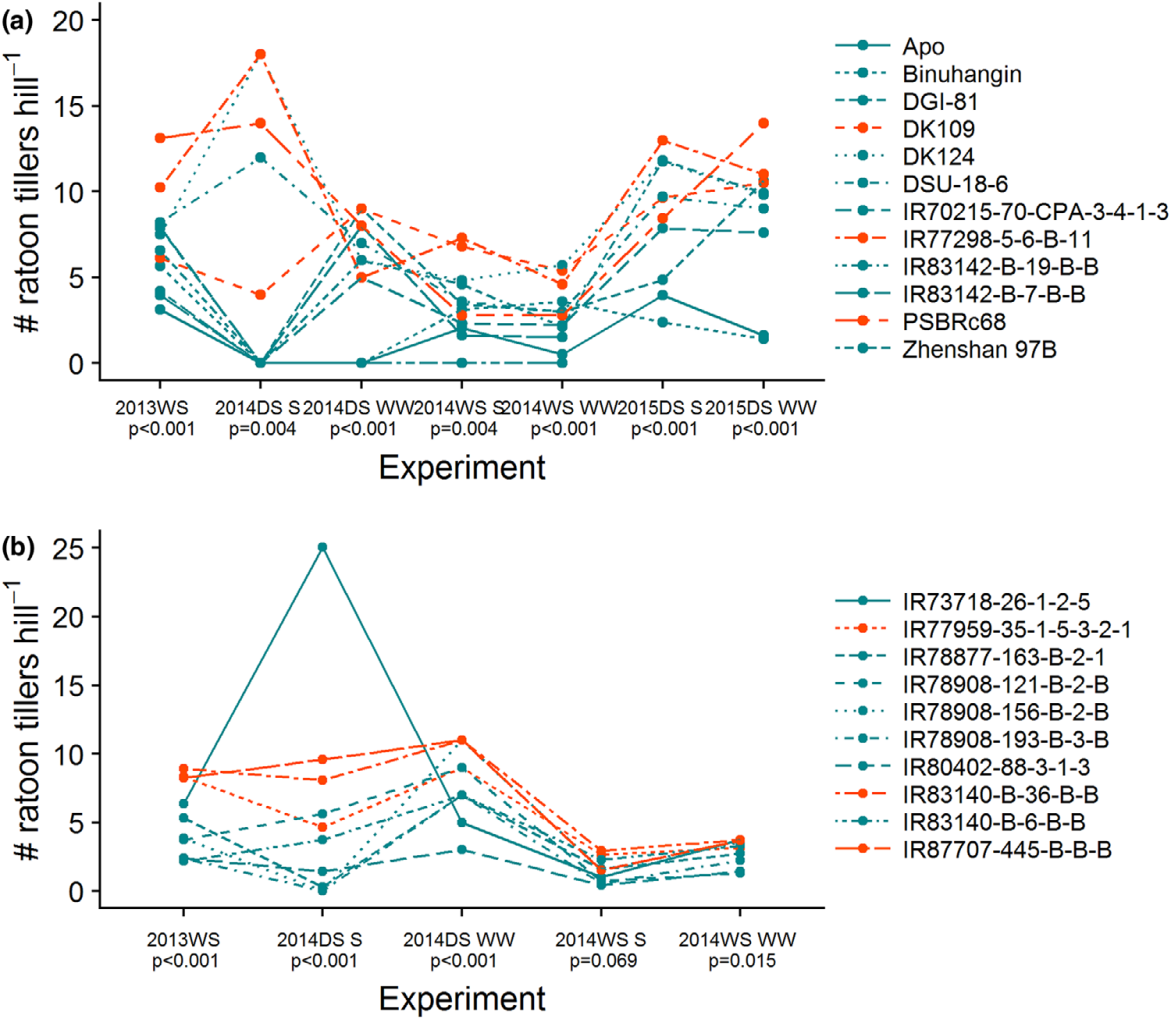
Postharvest ratooning: genotypic differences in tillering among two sets of genotypes ([a] drought resistance set, and [b] ratooner set) after cutting the mature main crop at a height of 20 cm. Tiller numbers were based on the final count in the plot (5–7 wk after main crop harvest). This multiseason assessment allowed for confirmation of the genotypes with the most consistent ratooning tillering ability (shown in orange), one of which (IR83140‐B‐36‐B‐B) was not initially classified as having high ratooning ability (Supplemental Table S1). DS, dry season; WS, wet season; S, drought stress treatment; WW, well‐watered treatment

The number of ratoon tillers in postharvest ratoon crops varied among genotypes (Supplemental Table S2) and generally reached a maximum around 35–40 d after harvest of the main crop (Supplemental Figure S3). The optimum soil moisture at the time of main crop harvest (at 30‐cm depth) for ratoon tillering was between 30 and 50% (by volume), and the numbers of ratoon tillers were reduced when the soil moisture levels were either too low or too high (Figure 2). The initial classifications of “low ratoon tillering” and “high ratoon tillering” genotypes were not completely consistent when repeated over multiple seasons in the present study, and the genotype × environment interaction for ratoon tiller number was significant (Supplemental Table S2). However, several genotypes were identified to produce consistently high numbers of tillers across seasons (Figure 3): PSBRc68, IR77298‐5‐6‐B‐11, and DK109 from the drought resistance set, and IR77959‐35‐1‐5‐3‐2‐1, IR83140‐B‐36‐B‐B, and IR87707‐445‐B‐B‐B from the ratooner set.

Postharvest ratoon tiller numbers increased when the main crop stubble height was increased from 5 to 20 cm (Supplemental Figure S3). The ratoon crop tiller numbers were significantly related to main crop tiller numbers and grain yield only in the ratooner set at a cutting height of 5 cm (Supplemental Table S3); no significant relationships between main crop growth and ratoon crop tiller number were observed in the drought tolerance set at 5 cm or in either set of genotypes at the stubble cutting height of 20 cm.

**Figure 4:**
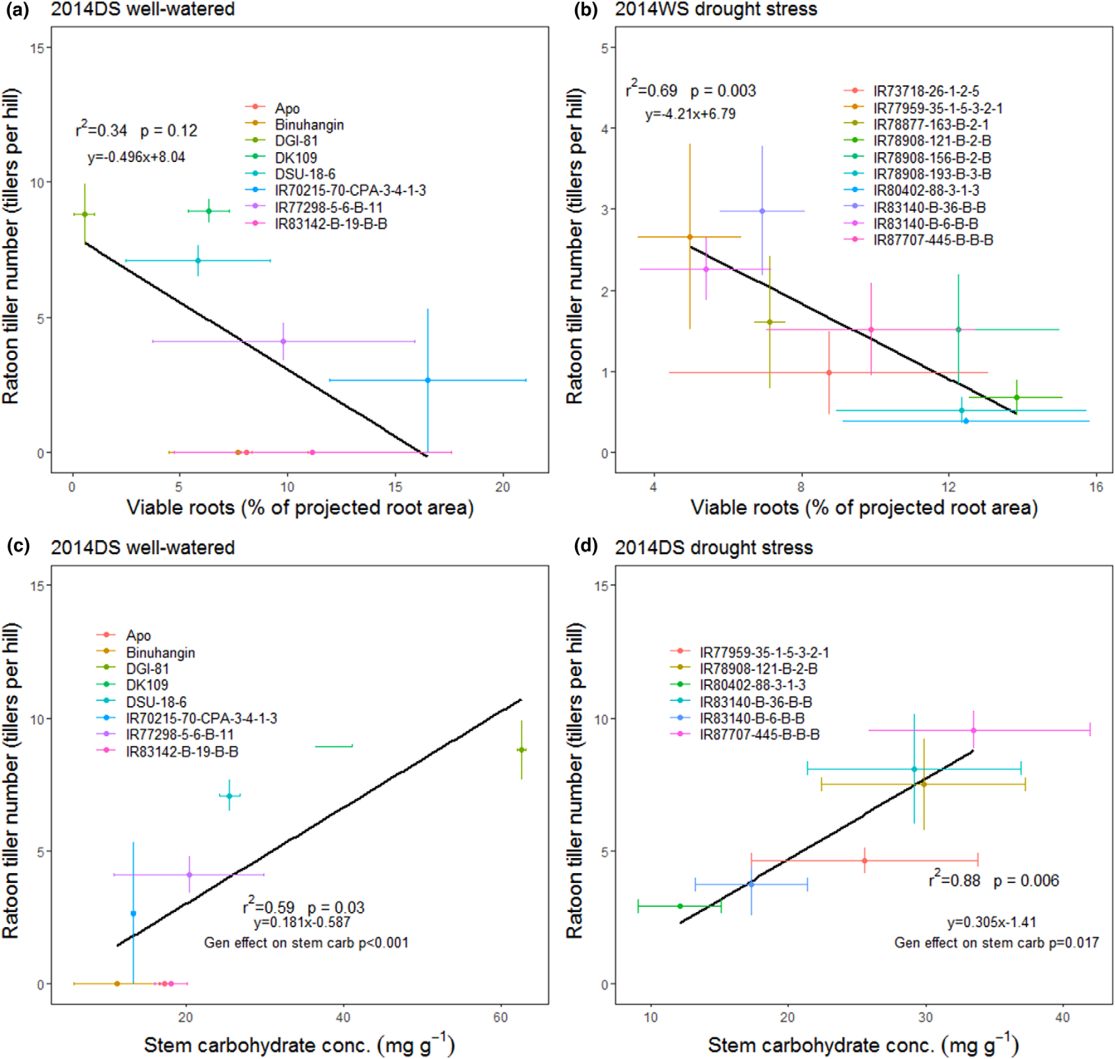
Postharvest ratooning: (a,b) measurements relating ratoon tiller growth with physiological parameters. Root viability in crown roots in 2014DS and 2014WS and (c,d) stem carbohydrate concentration of the main crop at harvest in the drought resistance set and the ratooner set in 2014DS. The ratoon tiller numbers shown are from the 20‐cm stubble cutting height. DS, dry season; WS, wet season

Although root viability at harvest did not vary significantly among genotypes (Supplemental Table S4), the root viability values in each plot were negatively correlated with ratoon tiller number in two experiments (Figure 4). Stem carbohydrate concentrations differed significantly among genotypes and ratoon tiller number increased correspondingly with stem carbohydrate concentrations across genotypes in the ratooner set drought treatment and in the drought resistance set well‐watered treatment (Figure 4), but not in the drought resistance set drought treatment (Supplemental Figure S4), which generally showed low stem carbohydrate levels.

### 3.2 Midseason ratooning: Lodged rice crops

**Figure 5:**
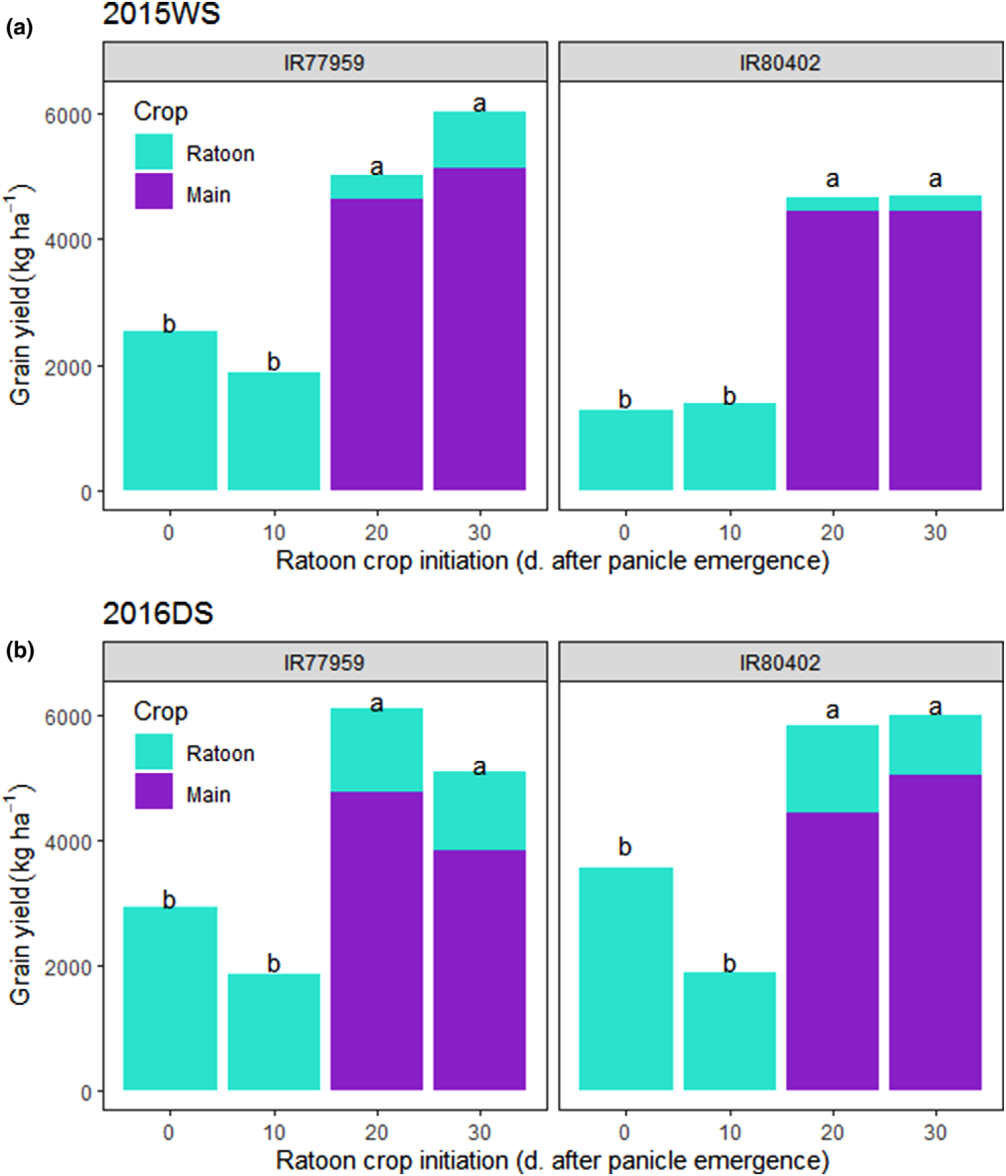
Total grain yield (kg ha^−1^) from the main crop and ratoon crop of each genotype in the 30‐ and 40‐cm stubble height treatments in (a) 2015WS and (b) 2016DS. The higher proportion of ratoon yield compared with the main crop yield of nonlodged IR77959 in 2016DS was likely due to bird damage on the main crop. The effect of ratoon crop initiation date on total yield (main + ratoon) is indicated by letter groups (*p* < .05). DS, dry season; WS, wet season

In lodging treatments imposed at 0 and 10 DAPE, grain yield was only obtainable from the ratoon crops and the grain yield from main crops was zero (Figure 5). Grain yields of the main crops were obtained only when the lodging treatment was imposed at 20 and 30 DAPE. The maximum combined seasonal grain yield of the main and ratoon crops were obtained from main crops lodged at maturity (30 DAPE), in which the increase by ratooning ranged from 17.5 to 33.0% and from 5.6 to 19.6% in IR77959 and IR80402, respectively, in 2015WS–2016DS (Figure 5).

**Figure 6:**
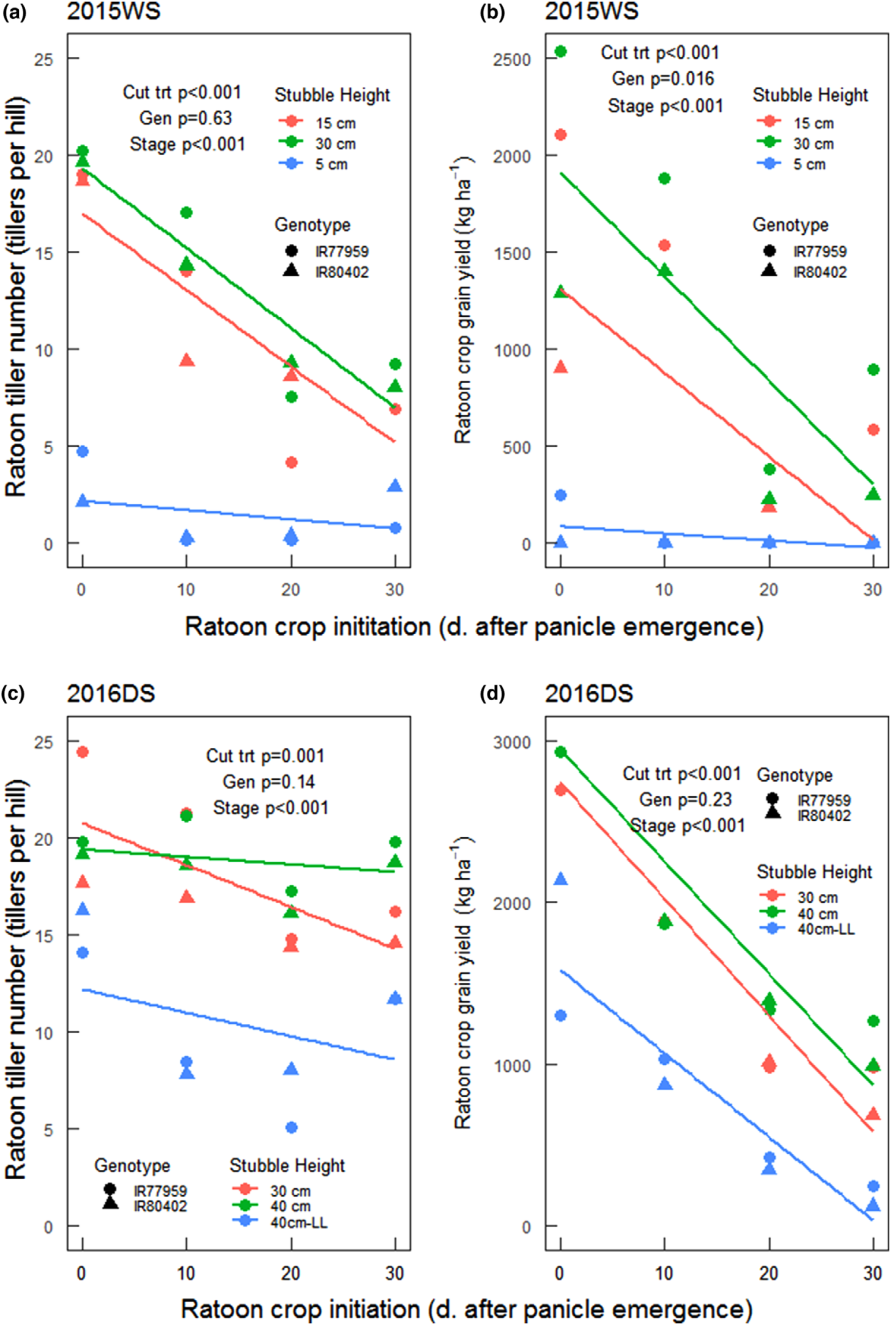
Midseason ratooning after lodging: ratoon tiller number and ratoon grain yield in the (a–b) 2015WS and (c–d) 2016DS well‐watered treatments as affected by main crop cutting height and time after panicle emergence at which the ratoon crop was initiated by mechanically lodging and removing the damaged main crop. The functions fitted to the data for the 5‐, 10‐, and 15‐cm cutting heights in Panel a were *y* = −0.04*x* +2.16, *y* = −0.39*x* + 17.0, and *y* = −0.41*x* + 19.3, and in Panel b were *y* = −3.68*x* + 85.9, *y* = −42.9*x* + 1307, and *y* = −53.5*x* + 1908. The functions fitted to the data for the 30‐, 40‐, and 40‐cm‐LL (lock lodged) cutting heights in Panel c were *y* = −0.22*x* + 20.7, *y* = −0.03*x* + 19.4, and in Panel d were *y* = −0.12*x* + 12.2, and y = −37.7*x* + 1764, and *y* = −40.8*x* + 1966, and *y* = −35.0*x* + 1120. DS, dry season; WS, wet season

Main crop grain yields were comparable between the two genotypes in both seasons except at maturity in 2016DS when IR77959 grains were damaged by birds. Grain yields of the ratoon crops were highest when the main crop was lodged earliest and in the taller stubble cutting height treatments (Figure 6, Supplemental Figure S5). The ratoon crop yields of IR77959 and IR80402 in treatments that were lodged at 0 DAPE were about 49.5–76.4% and 29.0–71.1%, respectively, of their corresponding nonlodged main crop yields at maturity.

Due to very poor tillering, almost no grains were harvested from the ratoon crop in the treatments with a stubble cutting height of 5 cm. When taller stubbles were used (30–40 cm), the highest ratoon crop grain yields were obtained from the crops that were lodged and cut at 0 DAPE. The yields generally declined when the ratoons were established at later dates after panicle emergence. The highest ratoon grain yields of IR77959 and IR80402, respectively, were 2.53–2.93 and 1.28–3.58 t ha^−1^ in 2015WS–2016DS. The lowest ratoon grain yields in 2016DS were obtained from the treatment with lock‐lodged stubbles (Figure 6, Supplemental Figure S5). Ratoon tiller number was significantly correlated with ratoon crop grain yield in the well‐watered treatments, but not in the drought stress treatment (Supplemental Table S3).

Similar to the trends observed in ratoon tiller numbers, ratoon crop grain yields were highest when the lodging or ratooning treatments were imposed at 0 DAPE and when taller stubble cutting heights were used and declined when lodging or ratooning was imposed at later crop stages and when stubble cutting heights were shorter (Figure 6). The two genotypes had comparable numbers of ratoon tillers in both seasons. In 2015WS, the ratoon tillers were generally taller when the ratoon crop was established either at 0 or 10 DAPE than at later crop stages (Supplemental Table S5). The ratoon tiller height was generally lower when grown from shorter stubbles. In 2016DS, the tillers from 40‐ and 30‐cm stubbles had comparable heights except when the stubbles were lock‐lodged, which resulted in lower tiller numbers. Stem carbohydrate concentrations in the stubble after lodging and cutting of the lodged shoots in 2016DS were highest at 10 and 20 DAPE (Supplemental Figure S6), which is a later peak than that of the ratoon tiller numbers and thus did not correlate with ratoon tiller numbers.

Ratoon crop durations were considerably shorter than the main crop durations (Supplemental Figure S7). The main crops of both varieties matured in 119 and 111 DAS in 2015WS and 2016DS, respectively, and the ratoon crops matured in >70 d when they were established at 0 DAPE and grown close to the ground either by short stubble cutting or by lock lodging (Supplemental Figure S7). At 30‐ and 40‐cm stubble heights, ratoon crops matured in <60 d. The total field durations of the main and ratoon crops were <140 d when the ratoons were established with 40‐cm stubbles either at 0 or 10 DAPE and were >168 d when ratoons were established at 20 and 30 DAPE either by lock lodging or by using 5‐cm stubbles.

### 3.3 Midseason ratooning: Drought‐damaged rice crops

**Figure 7:**
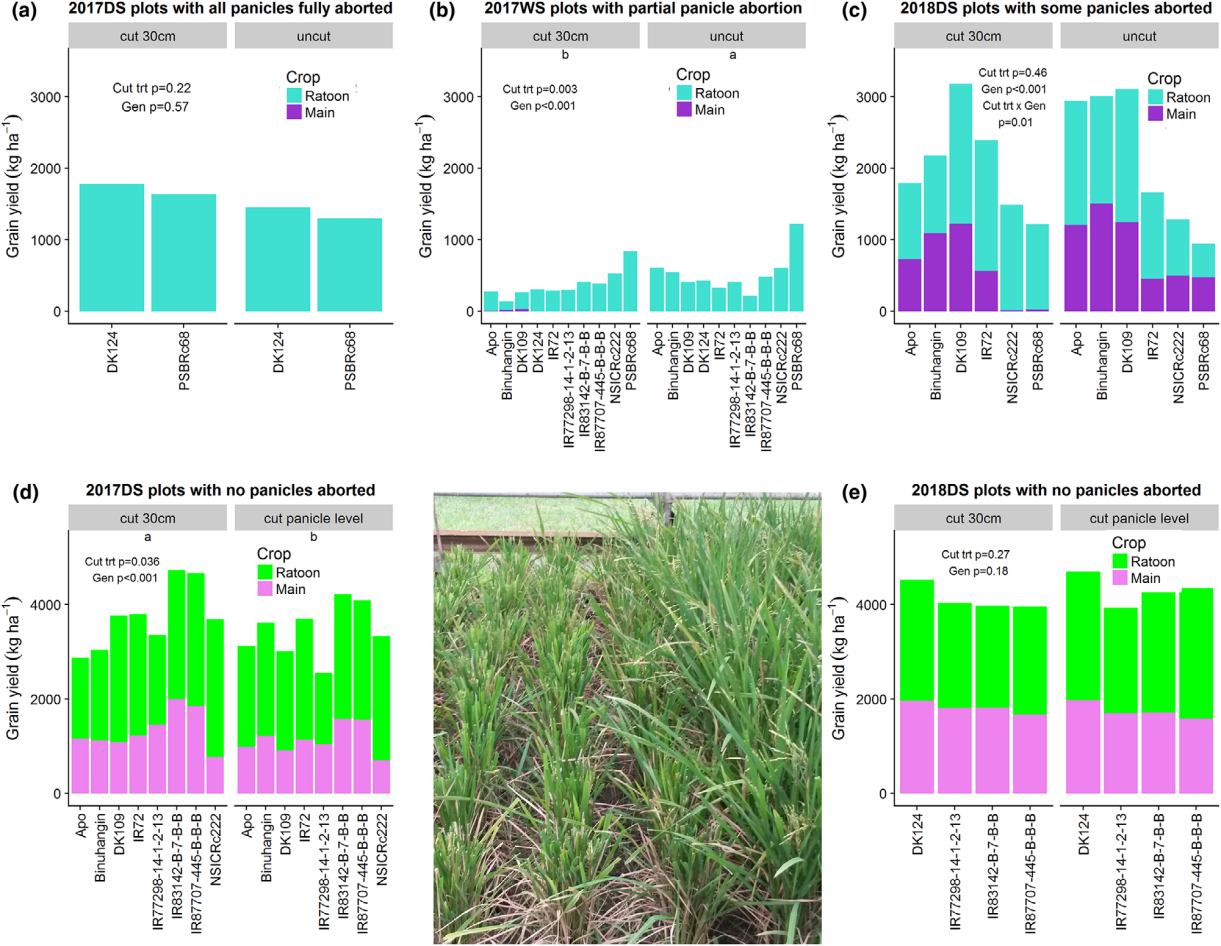
Midseason ratooning after drought stress: grain yield at the time of ending the drought stress treatment (“main crop”) and at maturity after rewatering and application of trimming treatments (“ratoon crop”). In plots showing panicle abortion due to drought stress (a–c), the trimming treatments were “cut at 30‐cm height” or “uncut.” In plots that did not show panicle abortion due to drought stress (d–e), the trimming treatments were “cut at 30‐cm height” or “cut at panicle level.” The *P* values shown indicate effects on total grain yield (main + ratoon crop). In cases where the effect of the cutting treatment was significant (b and d), letter groups are shown below the name of the cutting treatment to indicate which resulted in greater total grain yield values. The image shows the “cut at 30‐cm height” treatment on the left and the “uncut” treatment on the right after the drought stress treatment

The rate of progressive decline in soil moisture levels varied among the three drought experiments (Supplemental Figure S2). This variation in stress severity resulted in different degrees of panicle abortion due to drought in each experiment, to which the trimming treatments were modified accordingly. Generally, genotypes that flowered earlier tended to escape the drought stress and showed a lower degree of panicle abortion due to drought, and trials with less severe levels of drought stress showed less panicle abortion. The most consistent genotype showing panicle abortion due to drought was PSBRc68. In plots with panicle abortion, the trimming treatment had variable effects on the total grain yield (main crop + ratoon crop) across seasons and genotypes (Figure 7). In 2017DS, trimming the plants at a height of 30 cm produced greater total grain yield than trimming the plants at panicle level (Figure D). The trimming treatment significantly reduced grain yield of the plots with panicle abortion in 2017WS (Figure B). Genotypic differences in stem carbohydrate levels were observed in both trimming treatment and all three sampling dates after drought stress (Supplemental Table S4, Supplemental Figure S8). However, no correlations between grain yield and root viability or stem carbohydrate levels were observed in the drought ratoon experiments (Supplemental Table S4).

## 4 DISCUSSION

In this study, the type of crop damage with the most promising prospects for exploiting the ratooning ability of rice was midseason lodging. Without the ratoon tiller growth facilitated by cutting and removing the lodged stems, no grain yield could be harvested when lodging occurred early after heading. Ratoon growth in the drought stress experiments showed more variable results across different levels of drought severity and indicated that more research is necessary to identify under what specific types of drought leaf trimming treatments would be more likely to increase ratoon productivity. In terms of postharvest ratooning, results from this study suggest that genotype selection as well as well‐controlled soil moisture conditions at the time of main crop harvest could improve ratoon crop productivity.

We observed genotypic variation in the postharvest ratoon tillering ability of rice that was independent of preceding drought and well‐watered treatments and was not related to the level of drought tolerance (i.e., grain yield under drought) of the genotypes evaluated. However, it should be noted that the observed differences in postharvest ratoon tillering ability do not necessarily reflect differences in ratoon crop yield. Genotypes showing the highest levels of ratoon crop productivity included both relatively drought‐susceptible as well as drought‐tolerant released varieties based on previous evaluations (Torres & Henry, 2018). Interestingly, genotypes Binuhangin and IR87707‐445‐B‐B‐B (released as the variety DRR 42 in India) have both stood out as having high grain yield under drought in previous reports (Swamy et al., 2013; Torres, McNally, Vera Cruz, Serraj, & Henry, 2013) and have shown some similarities in their physiology in terms of low canopy temperature and deep root growth compared with other genotypes (Henry et al., 2015; Torres & Henry, 2018). However, this study revealed Binuhangin and IR87707‐445‐B‐B‐B to have much different ratooning ability, which was related to Binuhangin having much lower stem carbohydrate levels at the time of harvest, as observed in both the postharvest (Figure 4) and drought stress (Supplemental Figure S8) trials.

The relationship between stem carbohydrate levels and ratoon tiller growth was most evident in the postharvest ratoon trials (Figure 4), which is in agreement with previous studies in which stem carbohydrate levels were measured in relation to ratoon growth (He et al., 2019; Turner & Jund, 1993). In the midseason lodging experiments, it may also be implied that stem carbohydrates were an important factor in ratoon tiller growth based on the increased number of ratoon tillers with increased stubble cutting heights and earlier lodging or cutting dates (Figure 6). The lack of significant relationship between stem carbohydrate levels and ratoon tiller growth in the lodging trials may be due to the small number of genotypes evaluated that showed little variation in stem carbohydrate levels, which were only measured in the taller stubble cutting heights (Supplemental Figure S6). The role of stem carbohydrates in ratoon crop growth is also supported by previous reports of optimum main‐crop stubble cutting height for ratoon growth of 20–30 cm by Jones (1993) and of 15–20 cm by Bahar and De Datta (1977) and Chaterjee, Bhattacharya, and Debnath (1982), whereas Balasubramanian, Morachan, and Kaliapa (1970), and Bardhan Roy and Mondal (1982) observed no significant effect of stubble height on ratoon yield. In contrast with the lodging treatments with 30‐ and 40‐cm cutting heights, the number of ratoon tillers in the lock‐lodged subplots (cut at 40 cm) was very low (Figure 6, Supplemental Figure S5). These results imply that the subsequent waterlogging or loss of viability of stems in the lock‐lodged treatment impeded remobilization of stem carbohydrates to the developing ratoon tillers.

Despite genotypic differences in both ratoon crop productivity and stem carbohydrate levels in the drought experiments, these two factors were not correlated (Supplemental Table S4). In 2017WS, the lower level of ratoon crop productivity in the “cut at 30 cm” trimming treatments appears to be related to subsequently lower stem carbohydrate levels over time compared with the uncut treatment (Supplemental Figure S8), possibly due to reduced assimilation resulting from reduced (trimmed) leaf area. In contrast, plots with no panicle abortion in 2017DS showed that the trimming treatment at 30 cm was more beneficial than trimming at panicle level. Under well‐watered conditions, He et al. (2019) reported that ratoon tiller growth was reduced by leaf cutting treatments and promoted by panicle removal treatments. The difference in trimming results with the current study may be related to the response of rice to drought stress. Rice plants show notably high drought‐induced levels of stem carbohydrate remobilization among crops (Slewinski, 2012), which may have affected the availability of stem carbohydrate levels to support ratoon tiller growth in the drought trials compared with the lodging trials. Furthermore, panicle abortion due to drought could affect apical dominance regulating the development of dormant tiller buds (the hormonal effects of which have been documented; e.g., Liu et al., 2011; Zha, Imran, Xu, Ding, & Wang, 2019) and may explain why panicle removal seemed to confer less of an advantage in our drought trials than in our lodging trials.

In addition to the role of stem carbohydrates, we hypothesized that increased root viability at the time of main crop harvest is related to higher levels of ratoon crop productivity. Previous studies have reported that late‐season levels of root function are correlated with continued shoot viability in rice (i.e., stay‐green; Hoang & Kobata, 2009; Meng, Wei, Li, Dai, & Huo, 2018), and we expected this trend in shoot growth to extend its effects to the ratoon season, but this was not observed in our experiments. Due to root senescence, crown root viability at harvest was generally very low and unexpectedly showed some negative relationships with ratoon tiller growth (Figure 4)—this trend may reflect a carbon cost of maintaining root viability that caused a reduction in ratoon tillering and should be further investigated. Furthermore, we assumed that lodged rice crops from which damaged stems were not cut and removed would exhibit complete crop failure due to waterlogging and decomposition, but future studies could be conducted to quantify ratoon growth from such abandoned crops for a direct comparison with the ratoon management strategies this study is suggesting.

Postharvest ratooning may be suitable for crop intensification in tropical areas where monocropping is practiced, or where there is a wide turnaround period such as a rice–upland crop sequence where excessive soil moisture hampers dry land preparation after the rice crop harvest. Our study suggests that genotype selection could improve postharvest ratoon crop productivity, and the high ratooning ability of IR87707‐445‐B‐B‐B implies that there is not necessarily a tradeoff between drought tolerance of the main crop and ratooning ability. Postharvest ratoon tillering ability can also be altered by other factors such as the harvesting period duration, which could affect the stem carbohydrate contents and soil moisture conditions at the time of ratoon tiller formation. The lack of grain yield encountered with the postharvest ratoon crops in this study suggests that genetic resistance to pests and diseases, which could build up starting from the main crop, would be a vital factor in postharvest ratooning. Ratoon rice suffers from insect pests and diseases more than the main crop and would act as an alternate host for perpetuation of the pest to the for the succeeding main crop (Khrisnamurthy, 1988). Furthermore, since no fertilizer was applied on the ratoon crops in this study, the maximum grain yields of 2.93 and 3.58 t ha^−1^ obtained from otherwise abandoned rice crops could be further increased by fertilizer application. Evatt and Beachell (1960) reported that ratoon rice yields can be increased with N levels of up to 75% of what was applied to the main crop. Turner and McIIrath (1988) reported that N application after the harvest of main crop consistently increased ratoon crop yields.

## 5 CONCLUSIONS

Our results indicate that a grain yield of >3.5 t ha^−1^ can be obtained from an otherwise abandoned lodged rice crop by ratooning, especially when lodging occurs at earlier reproductive stages. Genotypic variation in ratoon tillering ability was observed, as well as effects of the environmental conditions under which the ratoon crop was grown; these factors can be optimized to increase ratoon crop productivity. Main crops that lodged before maturity produced greater ratoon yields when stubbles were cut at 30‐ or 40‐cm height, which was likely related to a more abundant carbohydrate pool than when they were cut at ground level. In the case of midseason drought, the benefits of trimming a moderately stressed crop appeared to be genotype specific and should be further characterized to specific types of drought stress. These results suggest that a management strategy to exploit the ratooning ability of rice could be developed to improve yields when midseason crop damage such as lodging or drought occurs.

## Supporting information

Supplemental material is available online for this article.
