# Supplementary material for: Ratooning as a management strategy for lodged or drought‐damaged rice crops
Source: Crop Sci. 2020 Jan 29;60(1):367–80. doi: 10.1002/csc2.20007 (PMC7282670; doi:10.1002/csc2.20007)
Supplement: Supplementary file 1 — Supplemental material is available online for this article. [file CSC2-60-367-s001.docx]

**Torres et al.**

**Ratooning as a management strategy for lodged or drought-damaged rice crops**

Supp. Table 1. Initial classification of rice genotypes evaluated for ratooning ability in this study for the post-harvest ratooning experiments and the ratooning experiments of crops damaged by mid-season drought. The genotypes shown in gray were later reclassified as consistently highest ratoon-tillering based on the results of this study.

| A) Post-harvest ratooning experiments | | |  |
| --- | --- | --- | --- |
| Drought resistance set |  | Ratooner set |  |
| **Drought tolerant genotypes** |  | **High ratoon tillering** **genotypes** |  |
| Apo |  | IR73718-26-1-2-5 |  |
| Binuhangin |  | IR78877-163-B-2-1 |  |
| DGI-81 |  | IR78908-193-B-3-B |  |
| DK 109 |  | IR83140-B-6-B-B |  |
| DK 124 |  | IR87707-445-B-B-B |  |
| DSU-18-6 |  |  |  |
| IR70215-70-CPA-3-4-1-3 |  | **Low ratoon tillering** **genotypes** |  |
| IR83142-B-19-B-B |  | IR77959-35-1-5-3-2-1 |  |
| IR83142-B-7-B-B |  | IR78908-121-B-2-B |  |
| PSBRc68 |  | IR78908-156-B-2-B |  |
|  |  | IR80402-88-3-1-3 |  |
| **Drought susceptible genotypes** |  | IR83140-B-36-B-B |  |
| IR77298-5-6-B-11 |  |  |  |
| Zhenshan 97B |  |  |  |
|  |  |  |  |
| B) Ratooning experiments of crops damaged by mid-season drought | | |  |
| **Drought tolerant genotypes** |  |  |  |
| Apo |  |  |  |
| Binuhangin |  |  |  |
| DK109 |  |  |  |
| DK124 |  |  |  |
| IR77298-14-1-2-13 |  |  |  |
| IR83142-B-7-B-B |  |  |  |
| IR87707-445-B-B-B |  |  |  |
|  |  |  |  |
| **High-yielding genotypes** |  |  |  |
| NSIC Rc222 |  |  |  |
| PSBRc68 |  |  |  |
| IR72 |  |  |  |

Supp. Table 2. Genotypic differences in ratooning ability (in terms of ratoon tiller number) among two sets of genotypes (A; genotypes selected for drought tolerance, B; genotypes selected for ratooning ability) after cutting the mature main crop at a height of 5 cm. Main crop treatments are indicated as DS: drought stress, WW: well-watered.

1. Drought resistance set

| **Pvalue** | 0.0000 | | 0.0006 | | 0.0000 | | 0.0006 | | 0.0000 | | 0.0043 | |
| --- | --- | --- | --- | --- | --- | --- | --- | --- | --- | --- | --- | --- |
| **Season Treatment** | **2013WSDS** |  | **2014DS DS** |  | **2014DS WW** |  | **2014WS DS** |  | **2014WS WW** |  | **2015DS DS** |  |
| **Ratoon tiller number (hill^-1^)** | | | | | | | | | | | | |
| Apo | 1.85 | bc | 0 | b | 0 | d | 0.4 | d | 0.52 | b | 3.21 | bc |
| Binuhangin | 1.88 | bc | 0 | b | 0 | d | 1.92 | bcd | 4.38 | a | 0.4 | c |
| DGI-81 | 2.92 | bc | 0 | b | 4.12 | abcd | 2.85 | abcd | 0.95 | b | 2.56 | c |
| DK109 | 1.2 | bc | 2.52 | ab | 4.72 | abc | 4 | abcd | 1.5 | ab | 5.73 | abc |
| DK124 | 1.88 | bc | 10.43 | a | 5.35 | ab | 5.03 | ab | 1.55 | ab | 10 | ab |
| DSU-18-6 | 2.5 | bc | 0.28 | b | 1.95 | bcd | 4.7 | abc | 1.6 | ab | 6.86 | abc |
| IR70215-70-CPA-3-4-1-3 | 4.1 | b | 0 | b | 1.62 | bcd | 0.92 | cd | 0.17 | b | 6.12 | abc |
| IR77298-5-6-B-11 | 1.85 | bc | 4.67 | ab | 1.85 | bcd | 6.38 | a | 0.88 | b | 10.46 | a |
| IR83142-B-19-B-B | 1.38 | bc | 0 | b | 0.85 | cd | 1.65 | bcd | 0.8 | b | 5.75 | abc |
| IR83142-B-7-B-B | 0.65 | c | 0 | b | 1.82 | bcd | 0.68 | cd | 2.52 | ab |  |  |
| PSBRc68 | 9.75 | a | 10.57 | a | 6.72 | a | 1.88 | bcd | 1.7 | ab | 6.61 | abc |
| Zhenshan 97B | 1.78 | bc | 0 | b | 0 | d | 0 | d | 0 | b |  |  |

1. Ratooner set

| **Pvalue** | 0.0022 | | 0.0000 | | 0.0000 | | 0.0033 | | 0.6953 | |
| --- | --- | --- | --- | --- | --- | --- | --- | --- | --- | --- |
| **Season**  **Treatment** | **2013WS DS** |  | **2014DS DS** |  | **2014WS DS** |  | **2014DS WW** |  | **2014WS WW** |  |
| **Ratoon tiller number (hill^-1^)** | | | | | | | | | | |
| IR73718-26-1-2-5 | 3.23 | ab | 21.48 | a | 0.65 | b | 3.97 | ab | 1.1 |  |
| IR77959-35-1-5-3-2-1 | 6.08 | a | 2.95 | b | 3.1 | a | 2.7 | ab | 1.38 |  |
| IR78877-163-B-2-1 | 1.92 | b | 0.25 | b | 1.98 | ab | 1.98 | ab | 1.23 |  |
| IR78908-121-B-2-B | 1.35 | b | 0.12 | b | 0.75 | b | 2.48 | ab | 0.62 |  |
| IR78908-156-B-2-B | 2.2 | ab | 0 | b | 1.57 | ab | 5.22 | a | 1.57 |  |
| IR78908-193-B-3-B | 0.95 | b | 0 | b | 0.95 | b | 2.5 | ab | 1 |  |
| IR80402-88-3-1-3 | 0.78 | b | 0.45 | b | 0.5 | b | 0.67 | b | 1.1 |  |
| IR83140-B-36-B-B | 2.12 | b | 3.75 | b | 2.85 | a | 6.15 | a | 1.1 |  |
| IR83140-B-6-B-B | 0.65 | b | 3.02 | b | 0.77 | b | 3.62 | ab | 1.1 |  |
| IR87707-445-B-B-B | 3.05 | ab | 5.78 | b | 1.75 | ab | 5.1 | a | 0.77 |  |

Supp Table 3. Relationships between ratoon tiller number and A) main crop tiller number, B) main crop grain yield across treatments and seasons, and C) ratoon crop grain yield by Pearson correlation.

1. Main crop tiller number vs. ratoon crop tiller number

| **Set of genotypes** | **Cutting ht** | **Pvalue** | **Coef** |
| --- | --- | --- | --- |
| Drought Resistance | 20cm | 0.9694 | -0.0028 |
| Drought Resistance | 5 cm | 0.3953 | 0.063 |
| Ratooner | 20cm | 0.1723 | 0.1254 |
| **Ratooner** | **5 cm** | **0.0023** | **0.2752** |

| **Season** | **Pvalue** | **Coeff** |
| --- | --- | --- |
| 2013WS | 0.8243 | -0.023 |
| 2014DS | 0.0661 | 0.1884 |
| 2014WS | 0.9976 | -0.0003 |
| 2015DS | 0.0935 | 0.1722 |

1. Main crop grain yield vs. ratoon crop tiller number

| **Set of genotypes** | **Cutting ht** | **Pvalue** | **Coef** |
| --- | --- | --- | --- |
| Drought Resistance | 20 cm | 0.3524 | 0.0689 |
| Drought Resistance | 5 cm | 0.3159 | -0.0743 |
| Ratooner | **20 cm** | **0.0001** | **-0.3529** |
| **Ratooner** | **5 cm** | **0.0006** | **-0.3087** |

| **Season** | **Pvalue** | **Coeff** |
| --- | --- | --- |
| 2013WS | 0.7143 | -0.0378 |
| 2014DS | 0.0896 | -0.1742 |
| **2014WS** | **0.0021** | **0.3109** |
| 2015DS | 0.0632 | -0.1903 |

1. Ratoon crop tiller number vs. ratoon crop grain yield

| **Season** | **Treatment** | **Pvalue** | **r^2^** |
| --- | --- | --- | --- |
| 2015WS | WW | <0.001 | 0.69 |
| 2016DS | WW | <0.001 | 0.31 |
| 2016DS | DS | n.s. |  |

Supp. Table 4. Genotypic effects and correlations with ratoon tiller number for A) Root viability and B) stem carbohydrate concentration. Abbreviations for post-harvest ratoon experiment sets: DR: Drought Resistance, R: Ratooner

| 1. **Root viability** | |  | | | | |  | | | |  | | | |  |
| --- | --- | --- | --- | --- | --- | --- | --- | --- | --- | --- | --- | --- | --- | --- | --- |
|  |  | |  | |  | |  | | | | Correlation with ratoon tiller number | | | |  |
| Season | Set | | Trt | | days after main crop harvest | | Genotype effect | | | | r^2^ | p-value | |  |  |
| 2014DS | DR | | Drought | | 1 | | 0.628 | | | | 0 | 0.8 | | |  |
|  | DR | | Well-watered | | 1 | | 0.347 | | | | 0.2608 | 0.0768 | | |  |
|  | R | | Drought | | 1 | | 0.21 | | | | 0 | 0.858 | | |  |
| 2014WS | DR | | Drought | | 1 | | 0.748 | | | | 0.07 | 0.63 | | |  |
|  |  | |  | | 29 | | 0.3144 | | | | 0 | 0.668 | | |  |
|  | DR | | Well-watered | | 1 | | 0.0702 | | | | 0 | 0.9 | | |  |
|  |  | |  | | 27 | | 0.1486 | | | | 0.03 | 0.228 | | |  |
|  | R | | Drought | | 1 | | 0.176 | | | | 0.1476 | 0.014 | | |  |
|  |  | |  | | 28 | | 0.2489 | | | | 0.014 | 0.45 | | |  |
|  | R | | Well-watered | | 1 | | 0.1383 | | | | 0.03 | 0.225 | | |  |
|  |  | |  | | 27 | | 0.0030 | | | | 0 | 0.558 | | |  |
| 2015DS | DR | | Drought | | 1 | | 0.5025 | | | | 0 | 0.63 | | |  |
|  | DR | | Well-watered | | 1 | | 0.0028 | | | | 0.03 | 0.217 | | |  |
|  |  | |  | |  | |  | | | |  |  | | |  |
|  | Trt | |  | | days after ending drought trt | |  | |  | correlation with total grain yield | | | | | |
| 2017WS | uncut | | Drought | | 15 | | 0.7585 | |  | 0.05 | | | 0.133 | | |
|  | cut 30cm | |  | | 15 | | 0.3440 | |  | 0.07 | | | 0.103 | | |
|  |  | |  | |  | |  | |  |  | | |  | | |
| 1. **Stem carbohydrate conc.** | | | | | | | |  | | | |  |  |  |  |
|  | |  | | days after ending drought trt | |  | | Correlation with ratoon tiller number | | | |  |  |  |  |
| 2017WS | | Trt | |  | | Genotype effect | | r^2^ | p-value | | |  |  |  |  |
|  | | cut | | 0 | | 0.0435 | | 0 | 0.92 | | |  |  |  |  |
|  | |  | | 15 | | 0.0287 | | 0 | 0.68 | | |  |  |  |  |
|  | |  | | 50 | | 0.0261 | | 0.0124 | 0.613 | | |  |  |  |  |
|  | |  | |  | |  | |  |  | | |  |  |  |  |
|  | | uncut | | 0 | | 0.0003 | | 0 | 0.847 | | |  |  |  |  |
|  | |  | | 15 | | 0.0385 | | 0 | 0.92 | | |  |  |  |  |
|  | |  | | 50 | | 0.0516 | | 0.026 | 0.456 | | |  |  |  |  |

Supp. Table 5. Mean ratoon tiller height (cm) of two genotypes as affected by the stubble height and crop stage when ratoon crops were established over two crop seasons.

| **Stage** |  | **2015WS** |  |  | **2016DS** |  |
| --- | --- | --- | --- | --- | --- | --- |
|  | **5 cm** | **15 cm** | **30 cm** | **30 cm** | **40 cm** | **40 cm-LL** |
| **00DAPE** | 77 a | 83 a | 89 a | 77 | 78 | 70 |
| **10DAPE** | 79 a | 78 a | 80 b | 69 | 71 | 64 |
| **20DAPE** | 67 b | 70 b | 73 c | 68 | 71 | 62 |
| **30DAPE** | 66 b | 69 b | 81 b | 73 | 75 | 60 |
| **Mean** | 72 | 75 | 81 | 72 a | 74 a | 64 b |

Means in a column or row within a season followed by a similar letter are not significantly different at 5% alpha,


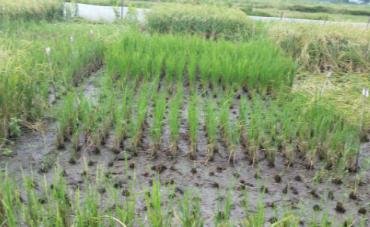


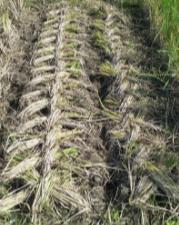

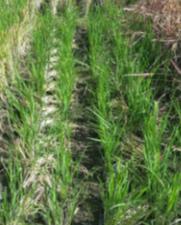


Supp. Fig. 1. Images from experiments on ratooning of lodged rice crops: A) stubble cutting heights from 5-40 cm were tested; B) the 40 cm stubble cutting height with lock-lodging treatment.


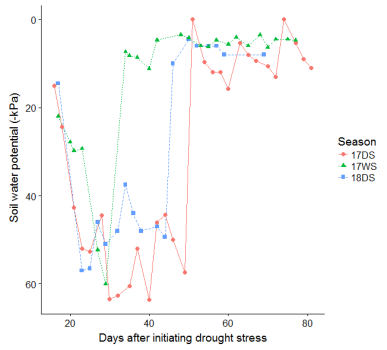


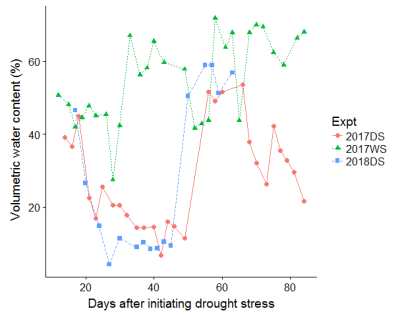


Supp. Fig. 2. Tensiometer (top) and volumetric soil moisture (bottom) readings at a soil depth of 30 cm during the three drought trials. Four to six tensiometers (Soilspec, TK Systems) were installed and 2-4 access tubes for monitoring soil moisture (Diviner 2000, Sentek) were installed in each drought trial.


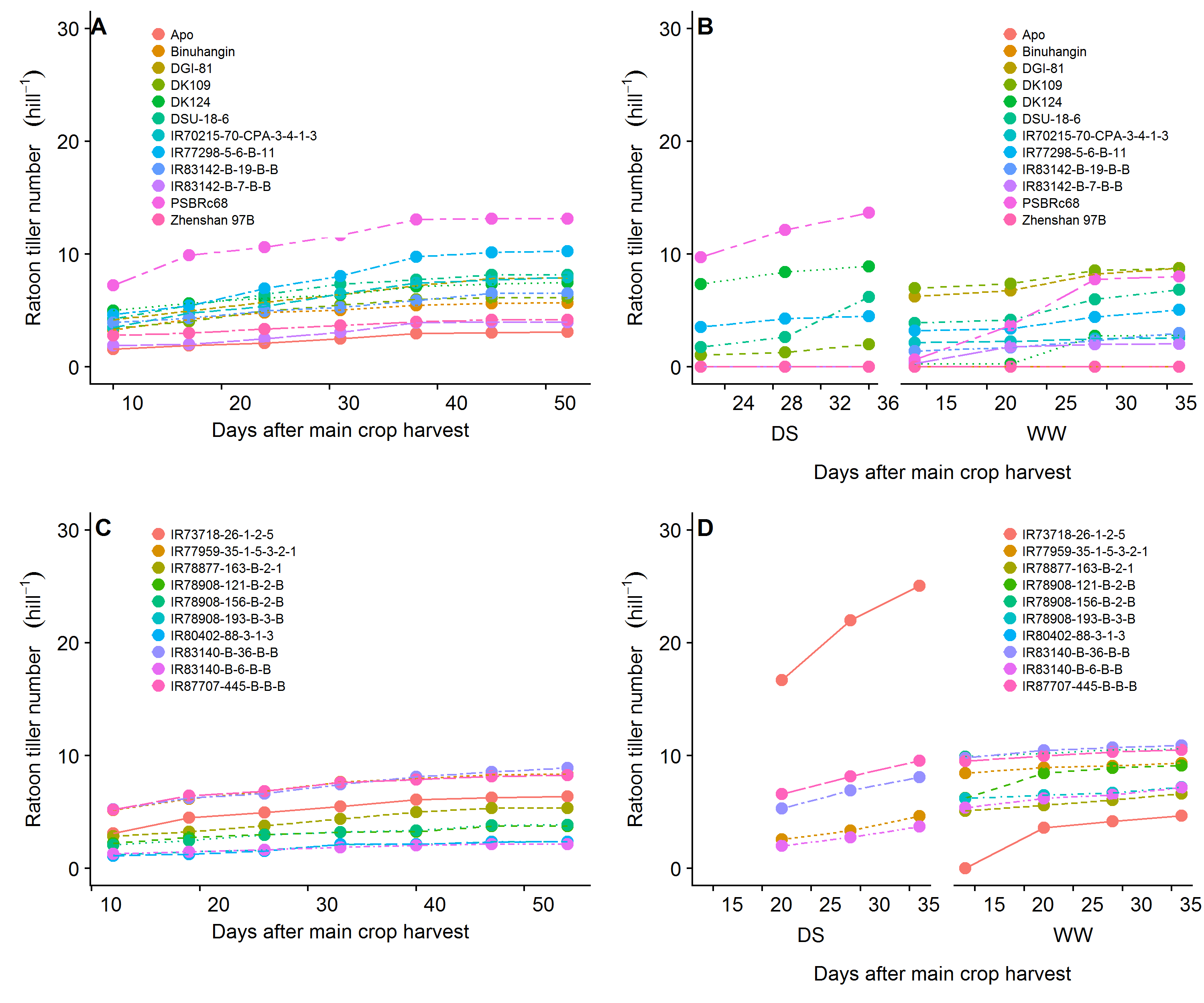


Supp. Fig. 3a. Post-harvest ratooning: Progression of ratoon tiller formation over time after harvest of the main crop, with a stublle cutting height of 20 cm. Drought tolerant set: A) 2013WS drought stress treatment, B) 2014DS; Ratooner set: C) 2013WS drought stress treatment, D) 2014DS.


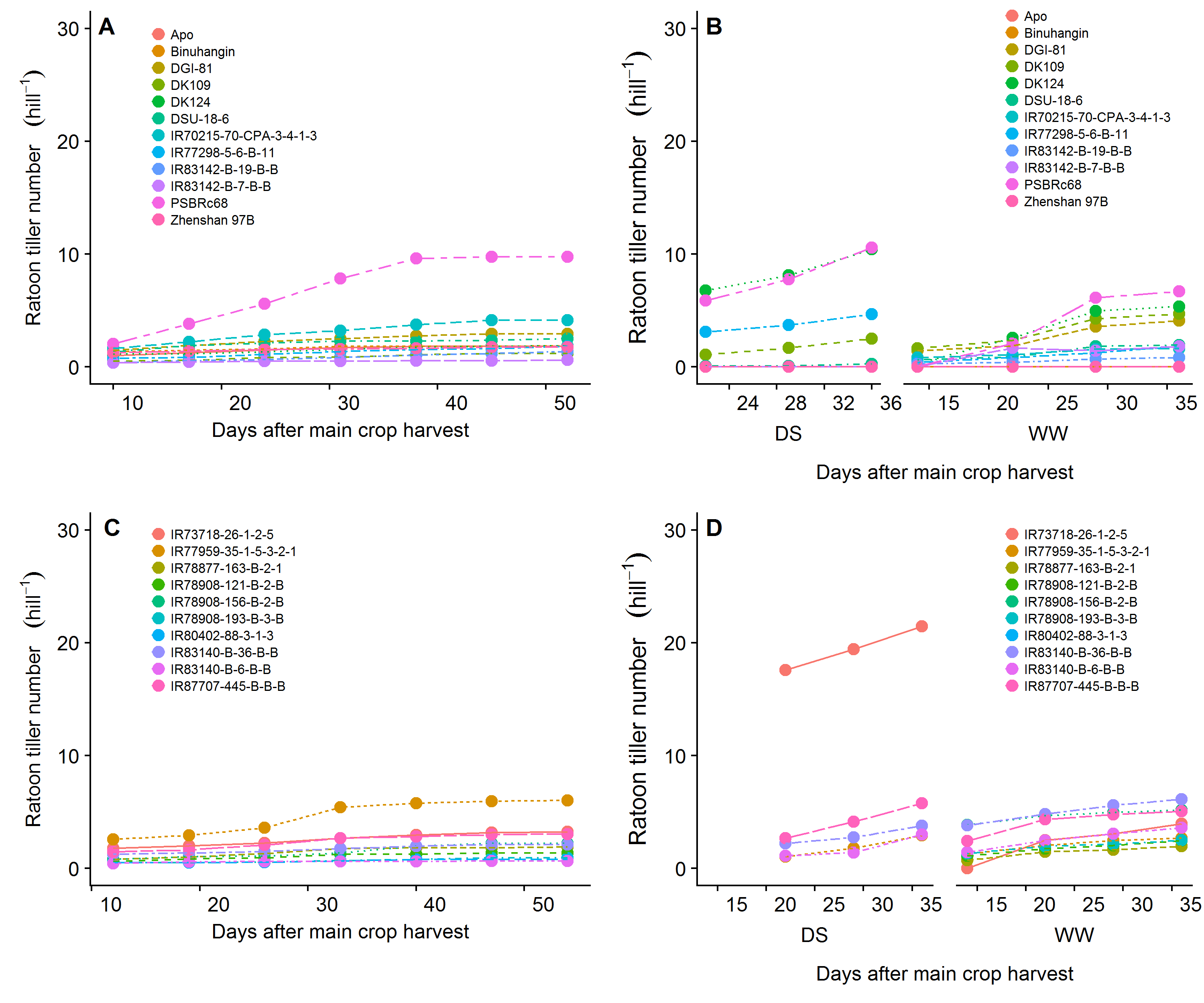


Supp. Fig. 3b. End of season ratooning: Progression of ratoon tiller formation over time after harvest of the main crop, at a stubble cutting height of 5 cm. Drought tolerance set: A) 2013WS drought stress treatment, B) 2014DS; Ratooner set: C) 2013WS drought stress treatment, D) 2014DS.


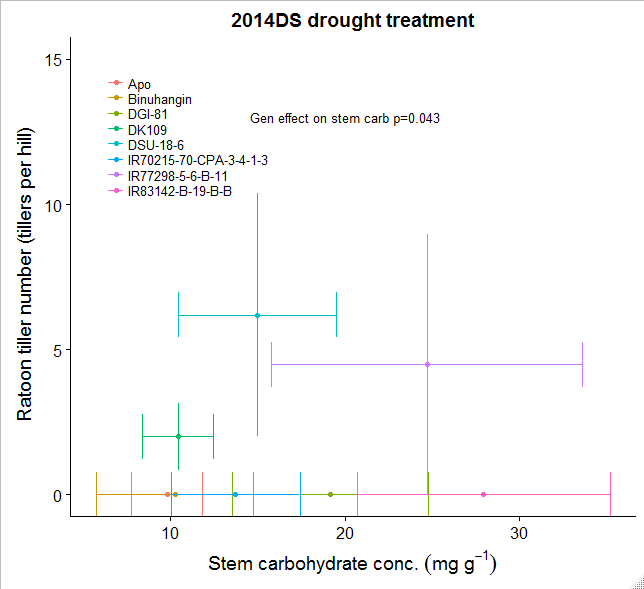


Supp. Fig. 4. Post-harvest ratooning: stem carbohydrate concentration of the main crop at harvest in the Drought Resistance set. The ratoon tiller numbers per hill shown are from the 20 cm stubble cutting height.


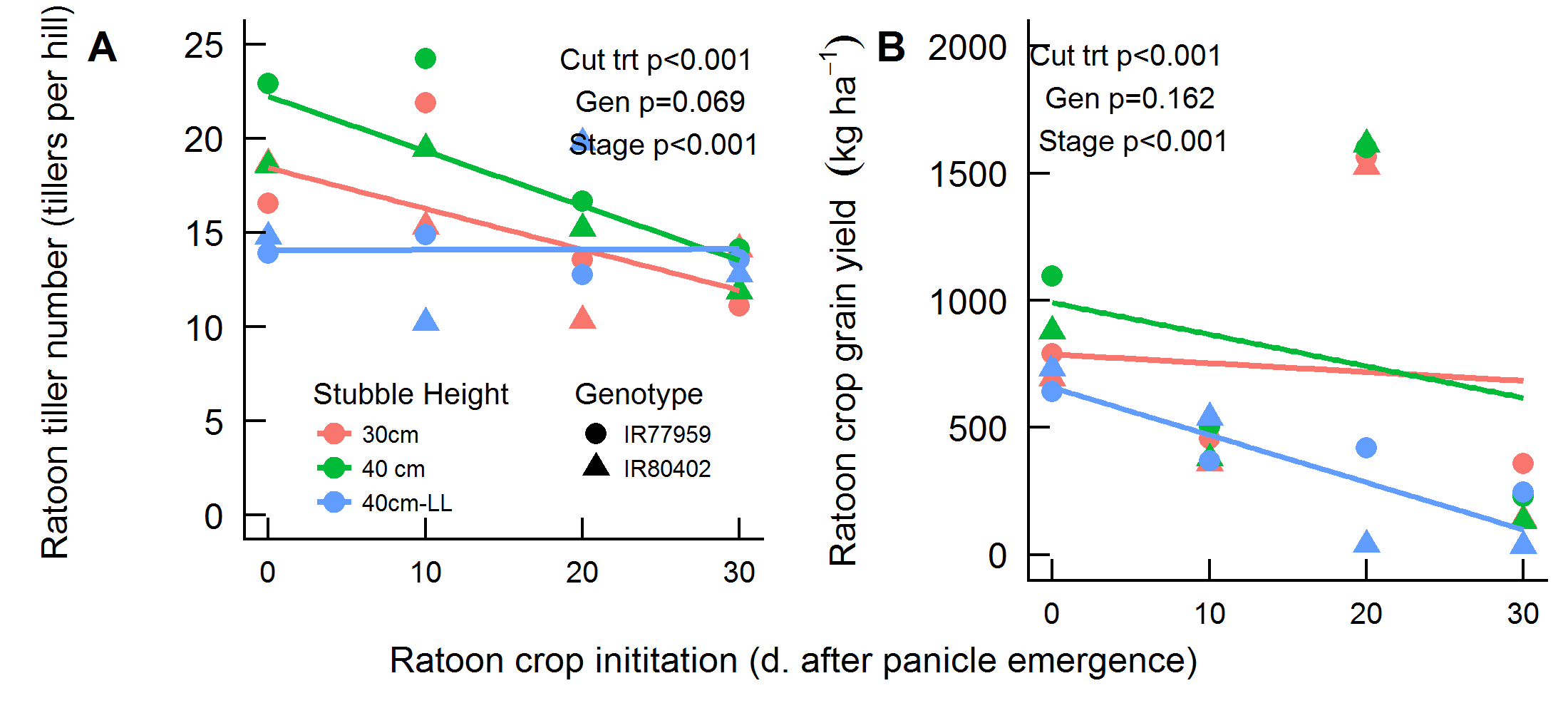


Supp. Fig 5. Mid-season ratooning following lodging: A) Ratoon tiller number and B) ratoon grain yield in the 2016DS drought stress treatment as affected by main crop cutting height and time after panicle emergence at which the ratoon crop was initiated by mechanically lodging and removing the damaged main crop. The functions fitted to the data for the 30, 40, and 40 cm-LL cutting heights in A) were y=-0.22+18.4, y=-0.16x+20.8, and y=0.002+12.1, y=-12.49x+991, and y=-18.6x+657, and y=-35.0x+1120, in B).


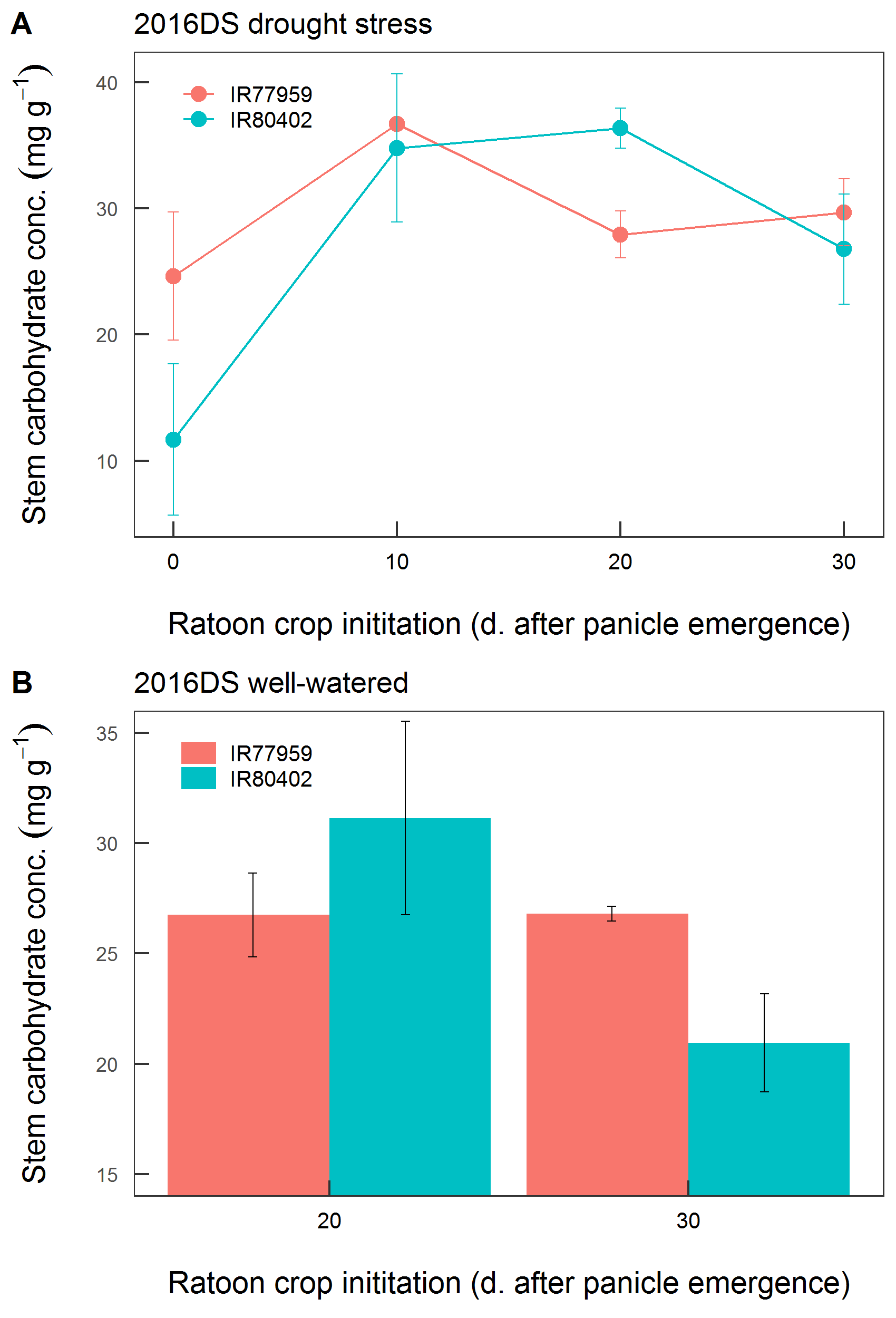


Supp. Fig. 6. Mid-season ratooning following lodging: Stem carbohydrate levels across lodging imposition dates in the 2016DS lodging experiment (A: drought stress treatment, B: well-watered treatment). Results are grouped from the 30 cm, 40 cm, and 40-LL stubble cutting height treatments.


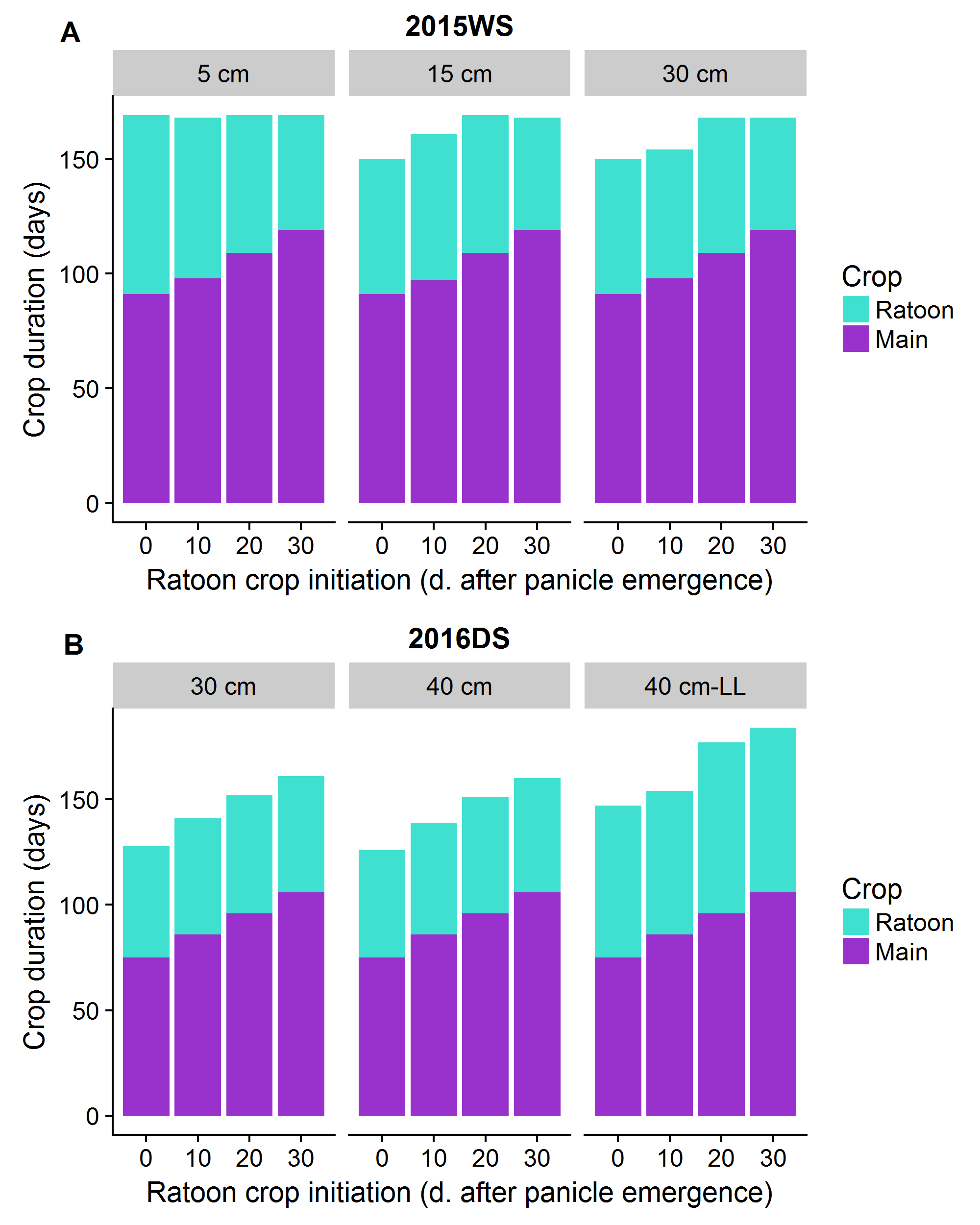


Supp. Fig. 7. Main, ratoon and total crop durations (days) of two genotypes as affected by the stubble height and crop stage when ratoon crops were initiated following lodging treatments.


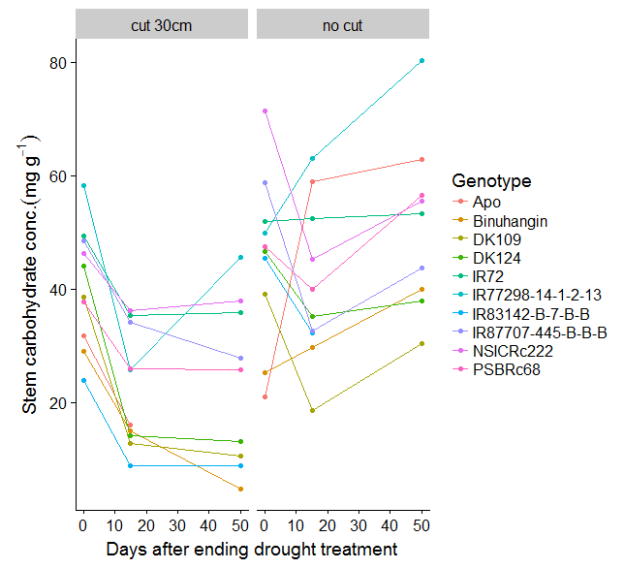


Supp. Fig 8. Stem carbohydrate concentrations over three sampling dates following drought stress in the “cut at 30 cm” or “uncut” treatments in 2017WS.
